# A generalized model for combining dependent SNP-level summary statistics and its extensions to statistics of other levels

**DOI:** 10.1038/s41598-019-41827-5

**Published:** 2019-04-02

**Authors:** Gulnara R. Svishcheva

**Affiliations:** 1 Institute of Cytology and Genetics, Siberian Branch of the Russian Academy of Sciences, Novosibirsk, 630090 Russia; 2 Vavilov Institute of General Genetics, Russian Academy of Sciences, Moscow, 119991 Russia

## Abstract

Here I propose a fundamentally new flexible model to reveal the association between a trait and a set of genetic variants in a genomic region/gene. This model was developed for the situation when original individual-level phenotype and genotype data are not available, but the researcher possesses the results of statistical analyses conducted on these data (namely, SNP-level summary Z score statistics and SNP-by-SNP correlations). The new model was analytically derived from the classical multiple linear regression model applied for the region-based association analysis of individual-level phenotype and genotype data by using the linear compression of data, where the SNP-by-SNP correlations are among the explanatory variables, and the summary *Z* score statistics are categorized as the response variables. I analytically show that the regional association analysis methods developed within the framework of the classical multiple linear regression model with additive effects of genetic variants can be reformulated in terms of the new model without the loss of information. The results obtained from the regional association analysis utilizing the classical model and those derived using the proposed model are identical when SNP-by-SNP correlations and SNP-level statistics are estimated from the same genetic data.

## Introduction

Over the past ten years, significant progress has been made in understanding human genetic variation and developing DNA reading technologies. Genome-wide association studies (GWAS) have emerged as a powerful tool for investigating the genetic architecture of complex traits. With the GWAS approach, a number of loci involved in the control of various complex traits, including diseases, have been identified. It turned out that most of the genetic variants associated with complex traits are located in noncoding regions of the genome, and their potential effects are associated with changes in the regulatory functions of the genome^1^. Unfortunately, the polymorphism of the loci identified to date can explain only a small fraction of the genetic variability of traits. This situation is typical of almost all complex traits^2^ and therefore the ‘missing heritability problem’ formulated several years ago^3–5^ remains the central issue of genetics.

One approach to finding missing heritability is by identifying rare genetic variants. Rare genetic variants with relatively large and therefore potentially recognizable effects are expected to contribute to almost all complex traits^4,6–8^. It is likely that these variants are located in the protein-coding regions and change the structure and function of the corresponding proteins. Recently, thanks to two scientific advances, it has become possible to identify rare genetic variants. One is large-scale exome sequencing, which allowed imputing a large number of missing genotypes using the reference data from ‘HapMap’^9^, ‘1000 Genomes’^10^, ‘HRC’^11^ and other projects. The other is а series of powerful statistical methods developed for regional association analysis (RAA) (for example^8,12–16^). The general principle of these methods is to simultaneously analyze all the rare genetic variants in a gene or a genome region (e.g. these belonging to a certain metabolic pathway). The family methods for RAA address a number of problems related to the low frequency of individual variants, multiple testing and interpretation of the results obtained, and increase the power of analysis^17^.

For analysis of regional associations, several model-based methods that use different regression models of trait inheritance have been developed. The main difference between these models is the assumption concerning the type of genotypic effects (fixed or random effects). In fixed-effects models, the immediate parameters of interest are genetic effect sizes. Models as these are used in the classical method of complete multiple linear regression analysis and its variants, when the genetic effect sizes or their specific linear combinations are estimated. Examples are as follows: collapsing methods^12,18–20^, principal component analysis methods^21^ and functional data analysis methods^22–26^. The random-effects models are based on the principle of decomposition of a total trait variance into components. These methods compare the genotypic and phenotypic similarities of individuals, and the parameter of interest is the component of trait’s variance that is explained by the genomic region. Under random-effects models, the kernel smoothing technique has proved to be successful^27–30^. This technique is based on the formation of a kernel matrix using the kernel smoothing density functions to measure the genetic similarity between individuals in the region. Methods exist that combine collapsing and variance components approaches^31^. In these methods, the parameters of interest are both the variance and the mean of the effect sizes. All the above RAA methods work with genotypic and phenotypic data measured for each individual (i.e. individual-level data).

To improve the accuracy of the analysis of specific traits, the results obtained from different samples can be combined using meta-analysis methods applied by various consortiums (for example, the International Consortium for Blood Pressure Genome-Wide Association Studies^32^. The Global Lipids Genetics Consortium genome-wide association studies^33^ and Genetics of Personality Consortium^34^). This makes it possible to increase the number of individuals involved in the analysis by up to several hundreds of thousands (see, for example^35^). Many effective approaches have been reported for gene-level meta-analysis of rare variants. The most popular ones are those based on burden^12,19^, SKAT and SKAT-O^36,37^ and also FLM tests^38–40^. It has been shown that meta-analysis significantly increases the power of GWAS^41,42^.

Recently, due to an increased emphasis on reproducibility and data sharing promoted by some journals and funding agencies, the SNP-level summary statistics obtained from GWAS have become increasingly available. Meta-analysis results, which are freely accessible, are usually presented with the size and significance of the effect of each genetic variant. Several methods have been developed to perform the RAA using summary statistics. These methods manipulate the p-values calculated for genetic variants within the region (Fisher method, p-value minimum method and others^43,44^). They have low power because the size and direction of the effect of each genetic variant are not taken into account. More powerful RAA methods using summary statistics have been developed that reproduce complete multiple linear regression (MLR) method^41^, collapsing (Burden) and variance components methods (SKAT and SKAT-O)^36^. For these methods, region-based statistics have been reformulated to use SNP-by-SNP correlations and SNP-level *Z* score statistics as input data instead original genotype and phenotype data.

However, for the inheritance of traits, regression models using summary statistics have not been developed, although such models can allow us to define dependencies between SNP-by-SNP correlations and SNP-level *Z* score statistics, to see how more adequately our theoretical assumptions can be implemented and give impetus to the development of new more powerful RAA methods. Moreover, it has not been shown that methods based on the principal components analysis (PCA) and functional linear models (FLM) can be reformatted for summary statistics.

Here I derive a common generalized model for combining dependent SNP-level summary statistics to perform a region-based association analysis between a single trait and a set of genetic variants of a genomic region. As data, the model utilises not only the summary SNP-level GWAS results for the trait of interest but also the SNP-by-SNP correlations estimated from the original genotype data or reference sample data. I analytically show that RAA methods developed for the classical linear regression model with additive effects of genetic variants can be reformulated within the new model without loss of information. The results (the p-values) of regional association analysis obtained using individual-level data and the proposed models are completely identical, when correlations between genetic variants and summary statistics are calculated from the same genetic data. Moreover, I analytically show that the proposed model can be extended to combine summary statistics obtained for other objects (genomic regions or traits) but using the same non-object-related data.

## Methods

### A single-trait model for individual phenotypic and genotypic data

#### On real data

For simplicity, suppose I have a sample of *n* unrelated individuals. For each individual, the phenotype and genotypes of *m* genetic variants (SNPs) in a genomic region are measured.

Consider the classical multiple linear regression model with additive effects, where the genotypes of the genetic variants are explanatory variables, and the phenotype is a response variable. This model can be written in a generalized form for the most popular model-based RAA methods as

1 $$y={e}_{n}\mu +GWC{\boldsymbol{\beta }}+{\xi }_{n}.$$

Here *y* is the (*n* × 1) known vector of continuous trait values; *G* is the (*n* × *m*) known matrix of SNP genotypes; *e*_*n*_ is the (*n* × 1) vector of *n* units; *μ* is the scalar intercept; *W* is the (*m* × *m*) diagonal matrix of weights assigned to SNPs (see **Box 1**); *С* is the (*m* × *k*) method-dependent matrix operator of the linear transformation of the weighted genotypes (see **Box 2**); *ξ*_*n*_ is the (*n* × 1) vector of random regression residuals, *ξ*_*n*_ is caused by an environmental factor and is supposed to be multi-normally distributed with a zero mean vector and the covariance matrix ${\sigma }_{y}^{2}{I}_{n}$, where ${\sigma }_{y}^{2}$ is the trait variance, and *I*_*n*_ is the identity matrix of order *n*; finally, ***β*** is the (*k* × 1) vector of regression coefficients measuring the effects of the *WС*-transformed genotypes on the trait.

It is standardly assumed that the trait *y* in Model (1) follows a multi-normal distribution with a mean vector *E*(*y*) and a covariance matrix *Cov*(*y*) determined in accordance with the type of genotype effects (fixed or random effects). For the fixed-effects (FE) models, $E(y)={e}_{n}{\mu }+GWC{\boldsymbol{\beta }}$ and $Cov(y)={\sigma }_{y}^{2}{I}_{n},$ where ***β*** is the vector of parameters of interest, concerning which the null and alternative hypotheses are formulated (H0: ***β*** = 0 against H1: ***β*** ≠ 0). For the random-effects (RE) models, *E*(*y*) = *e*_*n*_*μ* and $Cov(y)={{\boldsymbol{\tau }}}^{{\boldsymbol{2}}}(GWC{C}^{T}W{G}^{T})+{\sigma }_{y}^{2}{I}_{n},$ where ***τ***^**2**^ is the parameter of interest known as the trait variance component explained by the genomic region (H0: ***τ***^**2**^ = 0 against H1: ***τ***^**2**^ > 0).

### Box 1

#### Weights for SNPs

There are several weighting techniques. Since it is considered that rarer SNPs are more likely as causal variants with larger effect sizes, the SNP weights can be assigned inversely to their minor allelic frequency. One of such weighting techniques is simple thresholding, which filters genetic variants using the *c* threshold:

$$w(MA{F}_{i})=\{\begin{array}{cc}1, & if\,MA{F}_{i} > c,\\ 0, & if\,MA{F}_{i}\le c,\end{array}$$

where *w*() is the weight function and *MAF*_*i*_ is the minor allelic frequency of the *i*^th^ genetic variant.

Later, a continuous weighting technique was developed for kernel methods and it has become successful not only for the RE-model-based methods, but also for the FE-model-based methods. To calculate weights, the flexible beta density function has been proposed^29,30^:

$$w(MA{F}_{i})=\frac{MA{{F}_{i}}^{a-1}MA{{F}_{i}}^{b-1}}{B(a,b)},$$

where *B*(*a*, *b*) is the beta function with the pre-specified parameters *a* and *b*.

There is one more weighting technique using biologically functional information about genetic variants^27,28,37^. Using tools (for example, as PolyPhen2, SIFT, or RegulomeDB) the user can attempt to make a computational prediction of the functional impact of genetic variants and assign weights as the *a priori* probabilities of the functionality of the genetic variants.

### Box 2

#### Linear transformation of genotypes

The linearly transforming operator *C* can be defined in various ways depending on the RAA method used. In general, *C* has some constraints. For the FE-model, to avoid over-parameterization and multicollinearity, *C* must be a full rank matrix with a limited size (*m* × *k*) under condition that *n* ≥ *m* ≥ *k*. Moreover, to improve the model’s performance, it is advisable that the columns of *C* be orthogonal vectors (i.e. *С*^*T*^*C* = *I*_*k*_). For the RE-model, *C* must be such that *С*^*T*^*C* is able to be interpreted as a correlation matrix for effects ***β***.

Here I consider several examples for the most popular RAA methods. In the Burden method, *C* is an (*m* × 1) vector of units. It provides summation across the (weighted) genotypes of all the genetic variants into one vector. In FLM-based methods, *C* is an (*m* × *k*) matrix of values of *k* pre-specified basis functions (belonging to, for example, the Fourier basis or the B-spline basis) at *m* relative SNP positions and serves for functional (continuous) smoothing of the weighted genotypes. In PCA-based methods, *C* is given as an (*m* × *k*) incomplete (truncated) matrix of eigenvectors obtained from the spectral decomposition of the covariance matrix of the weighted genotypes. *C* serves to control the number of first principal components (*k*) involved in the analysis to cover 80–90% of total variance observed in the genomic region. In SKAT with a linear kernel, *C* is an identity matrix (*k* = *m*), and in SKAT-O with a linear kernel, *C* is a square root matrix of the matrix of correlations between genetic effects. It was introduced by Lee *et al*.^37^ as $C{C}^{T}=\rho {e}_{m}{e}_{m}^{T}+(1-\rho ){I}_{m},$ where *ρ* is a pairwise correlation among the genetic effect coefficients, *e*_*m*_ is the vector of units and *I*_*m*_ is the identity matrix of the *m-*th order.

#### On standardized data

For further notational convenience, I rewrite Model (1) into the standardized data format in accordance with H0, since GWAS SNP-level *Z* score statistics are calculated via the standardized (centered and scaled) *y* and *G*. Centering can be achieved through pre-multiplying all the terms of Model (1) by an (*n* × *n*) projection matrix, ${I}_{n}-\frac{{e}_{n}{e}_{n}^{T}}{n}$, while scaling can be performed by introducing a diagonal matrix *S* with diagonal elements ${s}_{ii}=\frac{{\sigma }_{y}}{{\sigma }_{{g}_{i}}},$ where *σ*_*gi*_ is the genotypic standard deviation of the *i*^th^ variant (see **Box 3**). In this way, I obtain a new regression equation

2 $$\bar{y}=\bar{G}{S}^{-1}WC{\boldsymbol{\beta }}+{\bar{\xi }}_{n}.$$

Here $\bar{y}$, $\bar{G}$ and ${\bar{\xi }}_{n}$ correspond to the standardized *y*, *G* and *ξ*_*n*_ in Model (1).

Note that the reformatting of Model (1) has no effect on *W*, *С*, ***β*** and, as will be shown below, on the region-based test statistic, since centering the data is reflected only in the intercept *μ* (*μ* = 0), and scaling the data leads to only the formation of the matrix *S*.

According to Model (2), the parameters of the distribution of $\bar{y}$ become $E(\bar{y})=\bar{G}{S}^{-1}WC{\boldsymbol{\beta }}$ and $Cov(\bar{y})={I}_{n}$ for fixed effects ***β***, and $E(\bar{y})={0}_{n}$ and $Cov(\bar{y})={{\boldsymbol{\tau }}}^{{\bf{2}}}(\bar{G}{S}^{-1}WC{C}^{T}W{S}^{-1}{\bar{G}}^{T})+{I}_{n}$ for random effects ***β***.

### Box 3

The matrix *S* represents an (*m* × *m*) matrix obtained by diagonalization of the vector of genotypic standard deviations divided by the standard deviation of the trait. The matrix *S* is caused by the scaling of phenotypes and genotypes and allows the regression coefficients to remain the same as in Model (1). Diagonal elements of *S* can be expressed via GWAS SNP-level beta standard errors, *se* (*β*_*GWAS*_):

$${s}_{ii}=\frac{{\sigma }_{y}}{{\sigma }_{{g}_{i}}}=\sqrt{n}se({\beta }_{GWA{S}_{i}}),$$

where the index *i* indicates the *i*^th^ variant.

### Single-trait tests using individual-level phenotype and genotype data

In the FE-model-based methods, tests based on the F distribution test statistics are often used:

3 $$F=\frac{n-1-r}{r}\frac{{R}^{2}}{1-{R}^{2}}.$$

Statistic (3) depends on the sample size (*n*), the maximum number of independent columns of the predictor matrix $\bar{G}{S}^{-1}WC(r=rank(\bar{G}{S}^{-1}WC))$ and the coefficient of determination (*R*^2^) calculated as

4 $${R}^{2}=\frac{1}{n}{\bar{y}}^{T}\bar{y}-\frac{1}{n}{\bar{\xi }}^{T}\bar{\xi }=1-\frac{1}{n}{(\bar{y}-\bar{G}{S}^{-1}WC{\boldsymbol{\beta }})}^{T}(\bar{y}-\bar{G}{S}^{-1}WC{\boldsymbol{\beta }}),$$

where the least square estimate of ***β*** maximizing *R*^*2*^ is ${({C}^{T}W{S}^{-1}{\bar{G}}^{T}\bar{G}{S}^{-1}WC)}^{-1}{C}^{T}W{S}^{-1}{\bar{G}}^{T}\bar{y}.$

The substitution of the estimate of ***β*** into (4) gives:

5 $${R}^{2}=\frac{1}{n}{\bar{y}}^{T}\bar{G}{S}^{-1}WC{({C}^{T}W{S}^{-1}{\bar{G}}^{T}\bar{G}{S}^{-1}WC)}^{-1}{C}^{T}W{S}^{-1}{\bar{G}}^{T}\bar{y}.$$

It followed that to avoid model over-parameterization and matrix non-invertibility problems in the FE-model, the condition *k* ≤ *m* ≤ *n* must be fulfilled, and the matrix product ${C}^{T}W{S}^{-1}{\bar{G}}^{T}\bar{G}{S}^{-1}WC$ in Exp. (5) should be a full rank matrix. However, if this is not the case, then additional regularizing procedures, which will not be considered here, are needed to achieve the invertibility of the matrix.

In the RE-model-based methods, tests based on a score test statistic, *Q*, are commonly applied:

6 $$Q=\frac{1}{n}{\bar{y}}^{T}K\bar{y},$$

where *K* is an (*n* × *n*) linear kernel matrix determined as $\frac{1}{n}\bar{G}{S}^{-1}WC{C}^{T}W{S}^{-1}{\bar{G}}^{T}$. The matrix *K* expresses the between-individual genetic similarity caused by the genomic region. Under the null hypothesis of no association, the distribution of *Q* is approximated by the weighted sum of ${\chi }_{1}^{2}$ distributions, where weights can be determined as eigenvalues of *K* denoted by *eigen*()^45^. Using the spectral decomposition property that the nonzero eigenvalues of *X*^*T*^*X* are the same as the nonzero eigenvalues of *XX*^*T*^, I have:

7 $$eigen(K)=eigen(\frac{1}{n}{C}^{T}W{S}^{-1}{\bar{G}}^{T}\bar{G}{S}^{-1}WC).$$

Thus, Model (2) is a linear regression model generalized for the popular RAA methods (Table 1) that assume the additive effects of genetic variants on the trait.

**Table 1:** Combined test statistic and parameters of its distribution using different types of initial data.

	Initial data			
	Individual phenotypes and genotypes		GWAS *Z* scores and SNP-by-SNP correlations	
Methods under FE-model	The coefficient of determination	The number of regression coefficients	The coefficient of determination	The number of regression coefficients
MLR	$\frac{1}{{n}_{0}}{\bar{y}}^{T}\tilde{G}{({\tilde{G}}^{T}\tilde{G})}^{-1}{\tilde{G}}^{T}\bar{y}$	$rank(\tilde{G})$	$\frac{1}{{n}_{0}}{z}^{T}{U}^{-1}z$	$rank(U)$
PCA/FLM	$\frac{1}{{n}_{0}}{\bar{y}}^{T}\tilde{G}WC{({C}^{T}W{\tilde{G}}^{T}\tilde{G}WC)}^{-1}{C}^{T}W{\tilde{G}}^{T}\bar{y}$	$rank(\tilde{G}WC)$	$\frac{1}{{n}_{0}}{z}^{T}{\tilde{S}}^{-1}WC{({C}^{T}W{\tilde{S}}^{-1}U{\tilde{S}}^{-1}WC)}^{-1}{C}^{T}W{\tilde{S}}^{-1}z$	$rank({C}^{T}W{\tilde{S}}^{-1}U{\tilde{S}}^{-1}WC)$
**Methods under RA-model**	***Q*** **test statistic**	**Eigenvalues of kernel**	***Q*** **test statistic**	**Eigenvalues of kernel**
Burden	$\frac{1}{{n}_{0}}{({e}_{m}^{T}W{\tilde{G}}^{T}\bar{y})}^{2}$	$\frac{1}{{n}_{0}}{e}_{m}^{T}W{\tilde{G}}^{T}\tilde{G}W{e}_{m}$	${({e}_{m}^{T}W{\tilde{S}}^{-1}z)}^{2}$	${e}_{m}^{T}W{\tilde{S}}^{-1}U{\tilde{S}}^{-1}W{e}_{m}$
SKAT-O	$\frac{1}{{n}_{0}}{\bar{y}}^{T}\tilde{G}WRW{\tilde{G}}^{T}\bar{y}$	$eigen(\frac{1}{{n}_{0}}RW{\tilde{G}}^{T}\tilde{G}W)$	${z}^{T}{\tilde{S}}^{-1}WRW{\mathop{S}\limits^{ \sim }}^{-1}z$	$eigen(W{\tilde{S}}^{-1}U{\tilde{S}}^{-1}WR)$
SKAT	$\frac{1}{{n}_{0}}{\bar{y}}^{T}\tilde{G}WW{\tilde{G}}^{T}\bar{y}$	$eigen(\frac{1}{{n}_{0}}W{\tilde{G}}^{T}\tilde{G}W)$	${z}^{T}{\tilde{S}}^{-1}WW{\tilde{S}}^{-1}z$	$eigen(W{\tilde{S}}^{-1}U{\tilde{S}}^{-1}W)$

*Notations*. *n*_0_: sample size minus one; *m*: number of SNPs in the region; $\bar{y}$: (*n* × 1) vector of standardized phenotypes; $\tilde{G}$: (*n* × *m*) matrix of centered genotypes; *W*: (*m* × *m*) diagonal matrix of SNP weights; for PCA method, *C*: (*m* × *k*) eigenvectors matrix obtained from the spectral decomposition of $W{\tilde{G}}^{T}\tilde{G}W$ or $W{\tilde{S}}^{-1}U{\tilde{S}}^{-1}W$ and truncated to *k* columns; for FLM method, *C*: (*m* × *k*) matrix of values of *k* basis functions at the relative SNP positions; *e*_*m*_: (*m* × 1) vector of units; *U*: (*m* × *m*) SNP-by-SNP correlation matrix; *R*: (*m* × *m*) matrix of correlations among genetic effects, $R=\rho {e}_{m}{e}_{m}^{T}+(1-\rho ){I}_{m},$ where *ρ* is an estimable parameter^37^; *eigen*(*X*): vector of eigenvalues of matrix *X*; *z*: vector of summary *Z score* statistics; $\tilde{S}$: (*m* × *m*) diagonal matrix with diagonal elements being equal as GWAS beta standard errors.

### A single-trait model for summary statistics

In this section, I am developing a new model for regional association analysis, where SNP-level summary statistics and SNP-by-SNP correlations are used as input data. The new model follows from Model (2), for which the linear compression of data is applied. As a result of this compression, the individual-level phenotypic and genotypic data are converted to the corresponding summary *Z* score statistics and correlations between the genetic variants.

I pre-multiply all the terms of Model (2) by an (*m* × *n*) compression matrix introduced here as $A=\frac{1}{\sqrt{n}}{\bar{G}}^{T}$:

8 $$A\bar{y}=A\bar{G}{S}^{-1}WC{\boldsymbol{\beta }}+A{\bar{\xi }}_{n}.$$

Two key points should be noted. First, the rank of the predictor matrix in Model (8) is the same as in Model (2), which means that the use of matrix *A* does not reduce the dimensional space of genotype data and, therefore, does not lead to loss of information. Secondly, $A{A}^{T}=\frac{1}{n}{\bar{G}}^{T}\bar{G}$ is an (*m* × *m*) SNP-by-SNP correlation matrix, therefore, $A{\bar{\xi }}_{n}$ represents a new random residual vector ${\bar{\xi }}_{m}$ with the distribution *N*(0, *U*), where $U=\frac{1}{n}{\bar{G}}^{T}\bar{G}.$

Thus, I obtain a new linear regression model from Model (8):

9 $$z=U{\tilde{S}}^{-1}WC{\boldsymbol{\beta }}+{\bar{\xi }}_{m}.$$

Here *z* is an (*m* × 1) vector of summary *Z* scores calculated in GWAS as $z=\frac{1}{\sqrt{n}}{\bar{G}}^{T}\bar{y},$ and $\tilde{S}=\frac{1}{\sqrt{n}}S$ is a diagonal matrix, whose diagonal elements are beta standard errors calculated in GWAS as ${\tilde{s}}_{ii}=\frac{{\sigma }_{y}}{\sqrt{n{\sigma }_{{g}_{i}}}}$ (see **Box 3**). In fact, the matrices ${\tilde{S}}^{-1}$ and *W* serve for weighting the SNP genotypes to control their impact on a trait of interest (for example, to increase the impact of rare variants), while the matrix *C* is method-dependent. *C* is set by the researcher in accordance with the selected gene-based method and serves for linear smoothing/compressing the SNP genotypes.

For better readability, Exp. (9) can be rewritten as

10 $$z=UX{\boldsymbol{\beta }}+{\bar{\xi }}_{m},$$

where $X={\tilde{S}}^{-1}WC$ is the (*m* × *k*) matrix that provides weighting and smoothing/compressing of the genetic data.

Thus, within the framework of Model (10), under the null hypothesis of no association, the vector *z* follows approximately a multivariate normal distribution as *N*(0, *U*), which was also shown in a work by Pasaniuc *et al*.^46,47^ and under the alternative hypothesis, *z* is distributed as $N(UX{\boldsymbol{\beta }},U)$ for the FE-model and as $N(0,{{\boldsymbol{\tau }}}^{2}(UX{X}^{T}U)+U)$ for the RE-model.

Like Model (2), Model (10) is based on the same standard assumptions as any linear regression with additive effects, namely: linearity and additivity of the relationship between dependent and independent variables, homoscedasticity and normality of the distribution of the regression residuals.

### Single-trait model-based tests using summary statistics

For RAA methods based on Model (10) with fixed effects, the *F* test statistic depends on the same parameters that describe the *F* test statistic (3) obtained from the original individual-level data: the sample size (*n*), the maximum number of independent columns of the predictor matrix $(r=rank(UX)=rank(\bar{G}X))$ and *R*^2^ reformulated from (5) as

11 $${R}^{2}=\frac{1}{n}{z}^{T}X{({X}^{T}UX)}^{-1}{X}^{T}z.$$

It is obvious that by analogy with ${C}^{T}W{S}^{-1}{\bar{G}}^{T}\bar{G}{S}^{-1}WC$ in Exp. (5), the full rank requirement should be generated only the matrix product ${X}^{T}UX$.

In RAA methods based on Model (10) with random effects, *Q* is calculated as

12 $$Q={z}^{T}{U}^{-1/2}{K}_{s}{U}^{-1/2}z,$$

where *K*_*s*_ is an (*m* × *m*) linear kernel matrix determined as ${U}^{1/2}X{X}^{T}{U}^{1/2}$. Here *K*_*s*_ expresses genetic similarity between ${U}^{-1/2}z$ statistics, which is based on the genetic correlations between individuals explained by the genomic region. The null distribution of *Q* from (12) is approximated by a weighted sum of ${\chi }_{1}^{2}$ distributions, where the weights are eigenvalues of *K*_s_:

13 $$eigen({K}_{s})=eigen({X}^{T}UX)$$

Note that the region-based test statistics *Q* (12) and (6) formulated in dissimilar terms are identical. Obviously, the new model (10) appears as a generalized model for the RAA model-based methods (Table 1), suggesting the additive effects of genetic variants on the trait.

So, Model (10) using summary statistics is based on the same standard assumptions as any linear regression with additive effects. However, when introducing Model (2), I also assumed that the sample consists of unrelated individuals, and the trait analysed is continuous. These restricting assumptions were made only for the sake of simplicity. They can be dropped within the framework of the new model, since for combining the computed Z scores, it does not matter what type of the trait is analyzed and what the structure of the sample is. This information is already taken into account when estimating the Z scores. For Model (10), it is primarily important that each Z score follows asymptotically a standard normal distribution, and correlations between these Z scores were known under H0.

### Expanding the model

To be able to exclude predictors from the model by shrinking their beta coefficients to zero, Model (10) can be expanded by using the lasso procedure, which is usually included for such purposes in the regression analysis (see, for example^48^). Since the estimates ***β*** and *R*^2^ in the new model are equivalent to those in the classical model using individual-level data, the regularization term,$\,\sum _{i=1}^{m}|{\beta }_{i}|$, added to the residual sum of squares (*RSS*) is the same in both models, because *RSS*=1 − *nR*^2^. Then the objective function to be minimized with respect to ***β*** is:

14 $$RSS+\lambda \sum _{i=1}^{m}|{\beta }_{i}|={(z-UX{\boldsymbol{\beta }})}^{T}{U}^{-1}(z-UX{\boldsymbol{\beta }})+\lambda \sum _{i=1}^{m}|{\beta }_{i}|$$

where *λ* is the tuning parameter. Without regularizing, the beta-coefficients are estimated as ${\boldsymbol{\beta }}={({X}^{T}UX)}^{-1}{X}^{T}z.$ However, the lasso regularization (14) has no analytical solution, and the numerical solution can be found by quadratic programming techniques from convex optimization and will not be considered here.

Thus, Model (10) is intended to combine a set of correlated SNP-level *Z* score statistics into a region/gene-level statistic. It covers all linear-regression methods with additive SNP effects treated both as fixed and as random. The required input data for Model (10) are, first, the *Z* score statistics measuring the associations of the same single trait with various SNPs, and, secondly, the correlations between the genotypes of these SNPs. GWAS SNP-level beta standard errors are optional input data for Model (10) because they are only required for the SNP weighting procedure.

It has been shown that the SNP-by-SNP correlations are asymptotically equal to the correlations between the Z scores at these SNPs under null data^46,47^, i.e. when $E[\bar{y}{\bar{y}}^{T}]={I}_{n},$

15 $$cor({z}_{1},{z}_{2})=E[\frac{{\bar{g}}_{1}^{T}\bar{y}}{\sqrt{n}}\,\frac{{\bar{y}}^{T}{\bar{g}}_{2}}{\sqrt{n}}]=\frac{1}{n}{\bar{g}}_{1}^{T}E[\bar{y}{\bar{y}}^{T}]{\bar{g}}_{2}=\frac{{\bar{g}}_{1}^{T}{\bar{g}}_{2}}{n}=cor({g}_{1},{g}_{2}),$$

where *z*_i_ is the *Z* score calculated as $\frac{{\bar{g}}_{i}^{T}\bar{y}}{\sqrt{n}}$ on the *i*^th^ SNP with the standardized genotype ${\bar{g}}_{i}\,$for the standardized trait $\bar{y}$. This makes it possible to turn from the specific to the general, i.e. to focus on any genetic objects, abstracting from SNPs. Then Model (10) can be interpreted in a new way:

16 $$z=UC{\boldsymbol{\beta }}+{\bar{\xi }}_{m}.$$

Here *z* denotes the vector of *Z* scores calculated at genetic objects, each of which is a genomic region (including a SNP and a gene), and *U* is the correlation matrix between these *Z* scores; the remaining variables are the same as in (10). In Model (16), the matrix of weights was removed, since it is unlikely that the researcher can formulate a hypothesis on the contributions of region-level *Z* score statistics to their combination. Such an interpretation (16) allows one to aggregate the region-level statistics, which were calculated for the same phenotype data (a trait or a set of traits), using the same RAA method, and then transformed into *Z* score statistics.

Moreover, since under null hypothesis the phenotypic correlations between traits are asymptotically equal to the correlations between *Z* scores calculated for these traits on the same genotype data^49^, namely

17 $$cor({z}_{1},{z}_{2})=E[\frac{{\bar{y}}_{1}^{T}\bar{g}}{\sqrt{n}}\,\frac{{\bar{g}}^{T}{\bar{y}}_{2}}{\sqrt{n}}]=\frac{1}{n}{\bar{y}}_{1}^{T}E[\bar{g}{\bar{g}}^{T}]{\bar{y}}_{2}=\frac{{\bar{y}}_{1}^{T}{\bar{y}}_{2}}{n}=cor({\bar{y}}_{1},{\bar{y}}_{2}),$$

it can be argued that Model (16) can be used to combine the trait-level statistics, which were obtained for various traits, using the same genotype data and the same method, and then transformed into *Z* score statistics. One confirmation of this is the score-based method, metaUSAT^50^, developed to find an association between a single genetic variant with multiple traits when using summary statistics. This method can be rewritten in terms of the new model with random effects. Then the *C* matrix in Exp. (16) can be formed by analogy with the SKAT-O method (**Box 2**) as $C{C}^{T}=\rho U+(1-\rho ){I}_{m}$, where *ρ* is a pairwise correlation among the genetic effect coefficients.

Note that to determine the association between a gene and a single trait, metaUSAT can be applied using the new model with non-unit weights for genetic variants. Obviously, the new regression model (16) includes a submodel that underlies the metaUSAT method.

Finally, I claim that Model (16) is suitable for aggregating test statistics calculated by various methods (for example, SKAT, SKAT-O, MLR, PCA and FLM) provided that the same original individual-level genotype and phenotype data were used and then, as usual, transformed into *Z* scores. Thus, Model (16) is universal because it is suitable for addressing tasks requiring that *Z* score statistics of any level be combined.

## Results and Conclusion

By using the technique that allows the linear compression of phenotype and genotype data to the level of statistics (namely, summary statistics and correlations among genetic variants) without the loss of statistical power, I have derived a new model for finding associations between traits and genomic regions. This model represents a linear regression model of fixed or random effects, where the vector-columns of the SNP-by-SNP correlation matrix serves as explanatory variables, and the vector of the SNP-level summary *Z* score statistics serves as a response variable.

Table 1 presents the exact formulas of the single-trait test statistic and the parameters of its distribution calculated by six popular RAA methods (MLR, PCA, FLM, Burden, SKAT and SKAT-O) based on the new model.

The methods built on the new model have a number of important advantages. First, these methods do not require access to individual-level phenotypes and genotypes, and use the SNP-level summary statistics obtained from the GWAS meta-analysis and deposited in public databases as input data. Secondly, these methods are fast, because most of the calculations have already been performed. Finally, these methods are universal, because they can combine dependent Z scores without requiring information on the population structure of the sample or the trait type. This information has been taken into account when calculating the summary Z scores.

In this work, I considered only the RAA methods that suggest an additive allele action in a genetic variant. For these methods, only the values of the SNP-level summary statistics and the correlations between genetic variants are required. Other methods that assume the dominant allele action can also be implemented under the new model using summary statistics. However, for such methods, higher-order SNP-by-SNP correlation matrices should be calculated. Noteworthy, the new model can serve as a basis for the development of new methods for conducting regional association analyses using SNP-level summary statistics and SNP-by-SNP correlations instead of individual measurements. Moreover, as has been shown in this work, the proposed model can be extended to combine Z score statistics derived from the test statistics with any level. In other words, the latter can be calculated for various genomic regions using the same individual phenotype data, for various traits using the same individual genotype data or by various methods using the same individual phenotype and genotype data.
